# CDK12: cellular functions and therapeutic potential of versatile player in cancer

**DOI:** 10.1093/narcan/zcaa003

**Published:** 2020-03-03

**Authors:** Kveta Pilarova, Jan Herudek, Dalibor Blazek

**Affiliations:** Central European Institute of Technology (CEITEC), Masaryk University, 62500 Brno, Czech Republic; Central European Institute of Technology (CEITEC), Masaryk University, 62500 Brno, Czech Republic; Central European Institute of Technology (CEITEC), Masaryk University, 62500 Brno, Czech Republic

## Abstract

Cyclin-dependent kinase 12 (CDK12) phosphorylates the C-terminal domain of RNA polymerase II and is needed for the optimal transcription elongation and translation of a subset of human protein-coding genes. The kinase has a pleiotropic effect on the maintenance of genome stability, and its inactivation in prostate and ovarian tumours results in focal tandem duplications, a CDK12-unique genome instability phenotype. *CDK12* aberrations were found in many other malignancies and have the potential to be used as biomarkers for therapeutic intervention. Moreover, the inhibition of CDK12 emerges as a promising strategy for treatment in several types of cancers. In this review, we summarize mechanisms that CDK12 utilizes for the regulation of gene expression and discuss how the perturbation of CDK12-sensitive genes contributes to the disruption of cell cycle progression and the onset of genome instability. Furthermore, we describe tumour-suppressive and oncogenic functions of CDK12 and its potential as a biomarker and inhibition target in anti-tumour treatments.

## INTRODUCTION

Cyclin-dependent kinase 12 (CDK12) was discovered as a candidate transcription and splicing machinery component by the Jonathon Pines lab in 2001 (1). Its heterodimer partner, cyclin K (CycK), was identified in a screen of proteins that can rescue the G1 progression defect and was associated with a strong kinase activity towards the C-terminal domain (CTD) of RNA polymerase II (RNAPII) (2). Nevertheless, it took until 2010 for the Arno Greenleaf lab to show that CycK and CDK12 are part of one complex functioning as an elongation-associated CTD kinase in *Drosophila* (3). This finding, together with discoveries that *CDK12* is among few recurrently mutated genes in ovarian carcinoma (4) and regulates genome stability via regulating the transcription of key DNA repair genes (5), sparked research interest in the cellular functions of CDK12.

**Figure 1.**
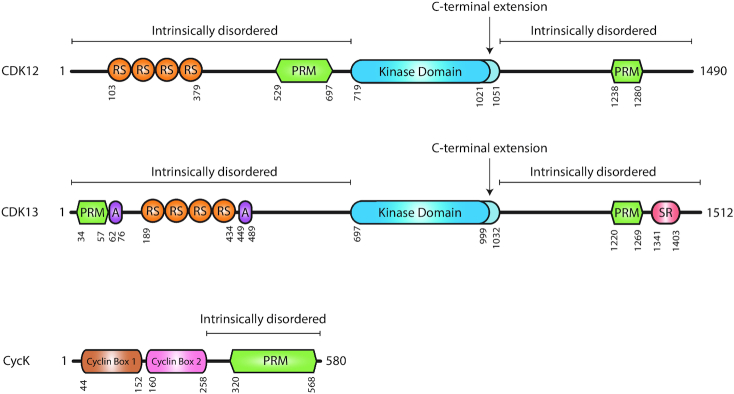
Domain composition and structure of human CDK12, CDK13 and CycK proteins. RS, PRM, SR and A correspond to arginine/serine-, proline-, serine- and alanine-rich domains, respectively. The kinase domains and their C-terminal extensions in CDK12 and CDK13 are indicated in dark and light blue, respectively. Numbers below the depictions indicate the amino acid position for a given domain. The regions of the proteins that are predicted to be intrinsically disordered are marked above their schemes.

Human CDK12 (also CRKRS, CRK7 or CRKR) is a 1490-amino-acid-long, ∼160-kDa protein consisting of a centrally located kinase domain and intrinsically disordered regions with N-terminal arginine/serine-rich (RS) and central and C-terminal proline-rich (PR) motifs (1,6) (Figure 1). The kinase domain contains a C-terminal kinase extension typical for CTD kinases involved in the regulation of transcription elongation (7). Its flexibility directs ATP binding (7–9) and is important for the catalytic activity of the kinase (7,10). CDK12 exerts its kinase activity only when associated with CycK, as documented by structural studies and similar changes in gene expression after depletion of the proteins (5,7). CycK is a ∼70-kDa protein consisting of two classical cyclin boxes mediating CDK12 association and a C-terminal extension rich in PR motifs with unknown functions (3,5,6,11,12) (Figure 1). Notably, CycK in higher metazoans also associates with CDK13, a kinase functionally distinct from CDK12 (5,8,13) despite the 93% sequence homology of their kinase domains (6). The functional differences are likely attributed to their N- and C-terminal extensions that are unusual for CDKs and whose sequences are largely different between both kinases (6) (Figure 1). *CDK12* and *CycK* are ubiquitously expressed in human tissues (1,2) and their null mice die at an early stage of development (5,14), indicating an essential role of the proteins in the adult as well as during development.

## CDK12 AS A CTD KINASE AND REGULATOR OF HUMAN GENE TRANSCRIPTION

RNAPII directs the transcription of protein-coding genes in a process consisting of initiation, promoter-proximal pausing, elongation and termination (15–18). It contains an unstructured CTD with 52 repeats of the evolutionarily conserved heptapeptide YSPTSPS, where individual serines (Ser2, Ser5, Ser7), threonine 4 (Thr4) and tyrosine 1 (Tyr1) are phosphorylated (19–21). The CTD modifications are necessary not only for the regulation of the transcription cycle, but also for the coupling of transcription with co-transcriptional processes such as splicing and 3′ end formation and processing (22–25). The phosphorylation of Ser5 (P-Ser5) marks RNAPII initiation and is required for establishing pausing (20,21). The phosphorylation of Ser2 (P-Ser2) is associated with active elongation and is needed for coupling transcription to mRNA processing. The transcription and CTD phosphorylation are regulated by several CDKs, but relatively little is known about the specific contributions of CDK12, although recent studies using novel experimental tools and genome-wide approaches have started to uncover some of its secrets.

### Role of human CDK12 in the phosphorylation of CTD

The role of human CDK12 in the phosphorylation of CTD remains controversial, as various experimental approaches provide different outcomes. This is further complicated by the well-known limitations of the use of phospho-CTD (P-CTD) antibodies (26), and by what seem to be different roles of human CDK12 and its homologues [Ctk1 in *Saccharomyces cerevisiae* (27,28), Lsk1 in *Schizosaccharomyces pombe* (29,30), CDK12 in *Caenorhabditis elegans* (31) and *Drosophila* (3)] in CTD phosphorylation. While the homologues are responsible for the majority of Ser2 modification in their species, their human counterpart is not, perhaps due to a partial mutual redundancy with other candidate P-Ser2 CTD kinases and elongation factors [CDK9 (32,33), CDK13 (8), CDK11 (Gajduskova *et al.*, in revision) and BRD4 (34)] on at least a subset of genes. This may complicate experimental conclusions in human cells.

Earlier studies in human cells indicated that longer term (days) CDK12 depletion leads to a modest decrease (3,5,11) or little change (13) in bulk P-Ser2. Distinct *in vitro* kinase assays showed that CDK12 can phosphorylate either Ser2 and Ser5 (5,7,8,11) or Ser5 and Ser7 (7). These experiments led to the conclusion that CDK12 is a promiscuous CTD kinase *in vitro* (7,35). Interestingly, the pre-phosphorylation of Ser7 further enhanced CDK12 kinase activity (7), while incubation with prolyl isomerase did not significantly affect it (8,35). The depletion of CDK12 coupled with ChIP-qPCR showed a decreased occupancy of P-Ser2 RNAPII at the 3′ ends of *c-MYC* and *c-FOS* genes (36,37).

Recent studies have used short (hours) CDK12-selective inhibition in cells (38–40) and P-CTD levels were measured with phospho-specific antibodies in total cell lysate. Covalent CDK12/CDK13 inhibitor THZ531 revealed either no changes in the bulk CTD phosphorylation with low doses (<100 nM) (38,40) or a decrease in P-Ser2 (38,40) and P-Thr4 (40) with higher doses (>200 nM). Inhibition with a competitive ATP analogue in analogue-sensitive (AS) CDK12 cell lines showed no strong changes in the bulk CTD phosphorylation, a slight decrease in P-Ser5 and especially P-Ser7, and a surprisingly slight accumulation of P-Ser2 (39,41). The differences in these findings can be explained either by off-targets of higher concentrations of THZ531 [CDK13, transcription-related JNK kinases (38) and perhaps other elongation kinases] or by a residual kinase activity in the presence of a competitive ATP analogue. Treatment with low doses (<100 nM) of novel competitive CDK12/CDK13 inhibitor SR-4835 results in a slight decrease in bulk Ser2 CTD phosphorylation (42). Since the low doses of THZ531, SR-4835 and inhibition of AS CDK12 kinase result in downregulation of a common subset of genes (DNA replication and repair genes) (38,39,42), it seems likely that the inhibition of human CDK12 causes relatively subtle changes in the CTD phosphorylation that are, however, critical for the optimal transcription of this subset of genes. Future experiments coupling short and CDK12-specific inhibition with mass spectrometric analyses of the CTD phosphorylation will likely provide more definitive answers to the conundrum of which residues and repeats in the CTD are modified by CDK12 in human cells (43,44).

### How do CDK12 perturbations affect gene expression?

In most studies, siRNA-mediated CDK12 depletion, the inhibition of AS CDK12 or low concentrations of THZ531 only led to expression changes in a subset of human genes (hundreds to thousands) rather than affecting global transcription (5,8,38–40,45–48). The optimal expression of long genes, mainly groups of DNA repair, replication and cell cycle genes, is particularly dependent on CDK12 (5,13,39,40,45,46). Notably, the depletion of CDK12 in three breast cancer cell lines led to differential expression of distinct genes; however, these cellular processes were confirmed to be commonly modulated by CDK12 in all three cell lines (46). CDK12 in *Drosophila* transcriptome has a gene-specific rather than global role, in which its targets include NRF2-dependent genes mediating oxidative stress response (49). CDK12 also counteracts the heterochromatin enrichment in the *Drosophila* X chromosome by blocking HP1 protein binding, predominantly affecting the expression of long neuronal genes (50).

### How does CDK12 regulate the transcription of its target genes?

**Figure 2.**
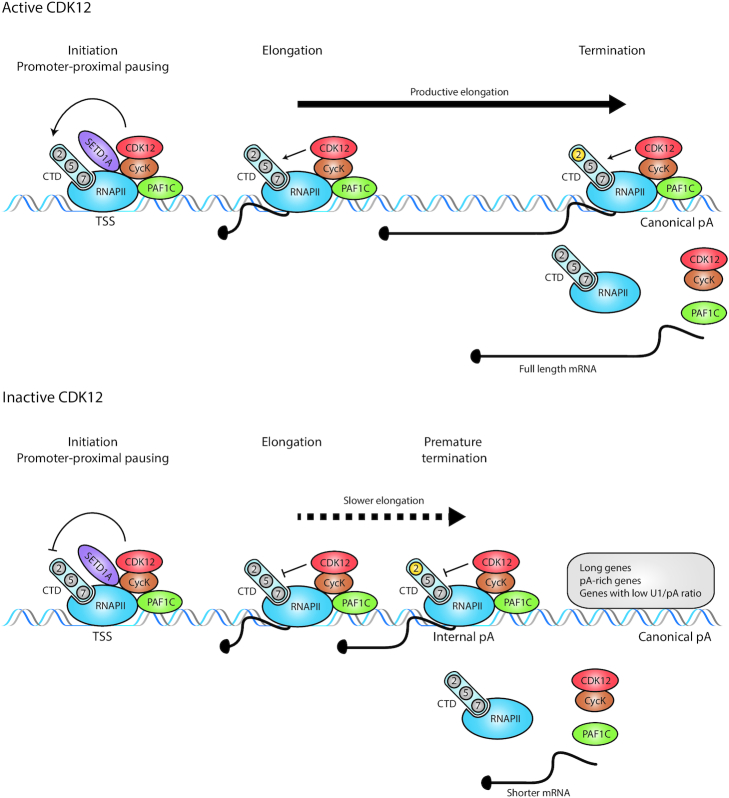
CDK12 kinase stimulates optimal transcription elongation and prevents premature termination of predominantly long, polyadenylation-site-rich genes. Working model: (top) the active CycK/CDK12 complex is recruited to the promoters (TSS) of genes by SETD1A and PAF1C proteins. CDK12 phosphorylates (thin black arrows) the CTD (light blue oval with two, five and seven in circles) of RNAPII, which results in productive elongation (thick black arrow) in gene bodies, the accumulation of P-Ser2 (2 in yellow circle) at the end of genes and optimal termination at canonical polyadenylation sites. Full-length mRNAs are produced. (Bottom) Upon CDK12 inhibition, the CTD is differentially phosphorylated (for simplicity not shown), which results in slower elongation (dotted black arrow) in the gene bodies of predominantly long, polyadenylation-site-rich genes with a low ratio of U1 snRNA binding to polyadenylation sites (U1/pA). P-Ser2 levels accumulate (2 in yellow circle) in the bodies of these genes approximately in the positions where RNAPII prematurely terminates (often at positions of internal cryptic polyadenylation sites). Shorter mRNAs are produced. pA = polyadenylation site.

Recent papers using different experimental models and novel tools have provided the first insights (Figure 2). Zhang *et al.* found CDK12 at promoters and gene bodies of protein-coding genes, largely overlapping with RNAPII occupancies (38), strongly supporting the idea that CDK12 travels with RNAPII and is an elongation-associated kinase (3). Inhibition with THZ531 led to a dose-dependent loss of RNAPII and its P-Ser2-modified forms from the 3′ ends of CDK12-sensitive genes, indicating an RNAPII elongation deficiency or termination defect (38). This was markedly different from an early elongation defect that blocks RNAPII release into gene bodies and is caused by CDK9 inhibition (32,51). It is worth noting that DNA repair genes were extremely sensitive to low THZ531 doses (<100 nM) at which bulk P-Ser2 levels were unaffected (38). The inducible depletion of CDK12 from mouse embryonic stem cells led to a global enhanced usage of intronic polyadenylation sites, resulting in the downregulation of full-length mRNA (protein) isoforms at the expense of shorter ones. Since homologous recombination (HR) DNA repair genes carry more intronic polyadenylation sites than other expressed genes, their cumulative usage in the absence of CDK12 gave a rationale for their unusual sensitivity to CDK12 loss (45). The inhibition of CDK12 by THZ531 or its AS form exhibited a gene-length-dependent elongation defect leading to a shortening of transcripts by premature termination in genes with a high number of cryptic polyadenylation sites, especially in DNA repair and core DNA replication genes (39,40). Lower GS content and a lower ratio of U1 snRNA binding to polyadenylation sites were identified as additional determinants of genes sensitive to CDK12 inhibition (40). In agreement, U1 snRNP was implicated in the regulation of transcription elongation and in preventing the premature termination of long genes enriched in DNA repair and cell cycle progression functions (52). Furthermore, SNRNP70, a U1 snRNP interacting factor, is phosphorylated in a THZ531-sensitive manner (40). Hence, further research of U1 snRNP will likely provide additional mechanistic understanding of CDK12 functions. The inhibition of AS CDK12 led to accumulation and shifts of RNAPII–P-Ser2 ChIP-seq peaks from 3′ ends to the bodies of individual CDK12-sensitive genes, approximately to the positions where transcription was lost and where premature termination occurred (39). This coincided with slower elongation rates in gene bodies (39) (Figure 2). Since there is known to be a reciprocal relationship between high P-Ser2 signal and efficient 3′ end processing (21,36), it will be important to determine the relationship between premature 3′ end processing induced by the CDK12 perturbations and the factors involved (including the relevant P-Ser2 kinase). A growing list of elongation modulators and factors regulating premature termination including PCF11 and SCAF4/8 will be a good starting point for these investigations (53,54). Of note, CDK12 inhibition impacted neither the recruitment of SPT6, a key elongation factor (39), nor noticeably the distribution of H3K36me3, an elongation-associated histone mark (Chirackal Manavalan, Kluge, Friedel and Blazek, unpublished data).

In summary, it appears that CDK12 prevents premature termination in the bodies of its target genes by facilitating their optimal elongation.

### How does CDK12 find its target genes?

Recent studies have provided the first suggestions to solve this conundrum (Figure 2). Yu *et al.* suggested that CDK12 is recruited by the PAF1C complex (55) (Figure 2), a critical regulator of pause release and elongation (56), to phosphorylate Ser2 in gene bodies (55). This model is consistent with the separate roles of yeast Ser2 kinases Bur1 and Ctk1 (CDK9 and CDK12 homologues), in mediating this modification at promoters and gene bodies, respectively (25,28). The PAF1C-mediated recruitment supports a role of CDK12 as a general transcription factor, but does not explain its gene-specific role in human cells. A recent study showed that PAF1C depletion leads to a gene-length-independent elongation defect together with an accumulation of P-Ser2 (and P-Ser5)-modified RNAPII in a 20–30-kb window downstream from transcription start sites (TSS) (57). This is reminiscent of the elongation defect in SPT5-depleted cells (58,59) and seems to be different from the accumulation of P-Ser2 predominantly at more distal regions of long CDK12-sensitive genes (39).

Hoshii *et al.* demonstrated that SETD1A, a histone methylase, recruits CycK to the promoters of DNA damage response genes (Figure 2) and regulates their expression in the S phase (60). This finding is consistent with CDK12 promoter occupancy (38) and its ability to regulate the transcription of DNA replication genes and G1/S progression (39). However, the functional relevance of the SETD1A-mediated recruitment of CDK12 to gene promoters for optimal elongation in the bodies of its target genes remains to be determined. Perhaps, this recruitment might also regulate the optimal CDK12-dependent release of promoter-paused RNAPII, as was observed on a few sample genes (39). Indeed, the depletion of SETD1A led to RNAPII accumulation at the selected target gene promoters (60).

Numerous studies found an association of CDK12 with mRNA processing components, including spliceosome factors, exon-junction complex (EJC) and other RNA-binding proteins (13,35,37,46). The functional significance of these interactions remains to be explored; however, the depletion of eIF4A3, an EJC component and CDK12 interactor, prevented the recruitment of CDK12 to the *c-FOS* gene and its 3′ end processing (61). Interestingly, an earlier study using a reporter system implied the ability of CycK to activate transcription via RNA (62). Whether nascent RNA (directly or indirectly) contributes to the recruitment of CycK/CDK12 to its target genes is an important question that remains to be answered.

## CDK12 IN mRNA PROCESSING (SPLICING, 3′ END PROCESSING)

The N-terminal 414 amino acids containing RS motifs are required for the localization of CDK12 into nuclear speckles (1,5,63,64), a nuclear subdomain enriched with splicing and 3′ end processing factors (65), indicating a role of CDK12 in mRNA processing (1,5,64). This observation was later substantiated by the identification of numerous core spliceosomal and SR proteins, hnRNPs, 3′ end processing factors, components of EJC and other RNA-binding proteins as CDK12 interacting factors (13,35,37,46). Some of the factors were also identified as candidate CDK12 substrates (40). Thus, CDK12 was suggested to link mRNA processing via a dual mechanism, partly by direct association of the elongating kinase with mRNA processing factors (1,13,35), and partly by indirect P-CTD-mediated (CDK12-dependent) recruitment of splicing and 3′ end processing components (66,67). Studies using reporters or individual genes indeed documented that CDK12 regulates the splicing of an E1a reporter minigene (63), serine–arginine splicing factor 1 (*SRSF1*) (13), glial-specific *neurexin IV* genes (68) and 3′ end processing of *c-MYC* and *c-FOS* (36,37). However, the depletion of full-length CDK12 coupled with a splicing-sensitive microarray or RNA-seq did not show any global splicing defects (5,45,46). Another study found a role of CDK12 in the modulation of alternative last exon (ALE) splicing, a specialized subtype of alternative mRNA splicing (46). The defect in ALE splicing appeared to be gene and cell type specific, affecting hundreds of longer, exon-rich genes with impacted proximal last exons containing multiple polyadenylation motifs (46). The mechanistic role of full-length CDK12 in the specificity of ALE splicing, which seems to be independent of CDK12-driven RNAPII processivity defects, remains to be determined (46).

The inhibition of CDK12 also did not produce any global splicing deregulation (38–40); however, an increase in the splicing efficiency of predominantly long genes was noted (40). This was determined to be an indirect consequence of an elongation defect and shortening of transcripts (39,40). Hence, it seems possible to conclude that CDK12 kinase activity (towards the CTD or other substrates) does not have a major direct role in the regulation of global pre-mRNA splicing. Notably, Ctk1 was also not reported to regulate splicing in *S. cerevisiae* (69). Although it lacks the RS domain, it binds the only three SR proteins present in yeast (35,70). Ctk1 depletion strongly reduces P-Ser2 and affects the co-transcriptional recruitment of the 3′ end processing factors without exhibiting any transcriptional defects (71–73). Likewise, inhibition of the fission yeast non-essential protein Lsk1 led to a marked decrease in P-Ser2 levels without a significant effect on transcription (29). Only a slight impact on elongating RNAPII was noted just past the 3′ end cleavage polyadenylation termination region, likely a consequence of inefficient recruitment of 3′ processing machinery (29).

## CDK12 IN TRANSLATION

**Figure 3.**
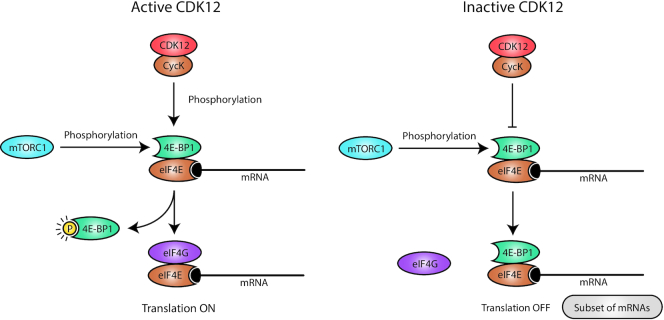
CDK12 regulates optimal translation of subset of mRNAs. (Left) CDK12 in collaboration with mTORC1 kinase phosphorylates the translational repressor 4E-BP1, which leads to its release from 5′ cap (black oval)-bound eIF4E. Subsequent recruitment of the eIF4G translation initiation complex to the eIF4E on the subset of mRNAs results in their efficient translation. (Right) CDK12 depletion results in a diminished phosphorylation of 4E-BP1, which stays bound to eIF4E and blocks the recruitment of eIF4G, which prevents the translation of the CDK12-specific subset of mRNAs.

Apart from its role in mRNA synthesis, CDK12 also regulates the translation of a subset (hundreds) of mRNAs (74) (Figure 3). In collaboration with mTORC1, CDK12 phosphorylates the translation repressor 4E-BP1 and controls the binding of translation initiation factor eIF4G, which facilitates the efficient translation of key subunits of centrosome, centromere and kinetochore complexes and also of CHK1, a key regulator of G1/S and G2/M progression (74). Hence, the role of CDK12 in translation in metazoans seems to be more specialized than its yeast Ctk1 homologue, which directs global translation during initiation steps as well as during elongation (75,76).

## CDK12 IN REGULATION OF CELL CYCLE PROGRESSION AND CELLULAR PROLIFERATION

**Figure 4.**
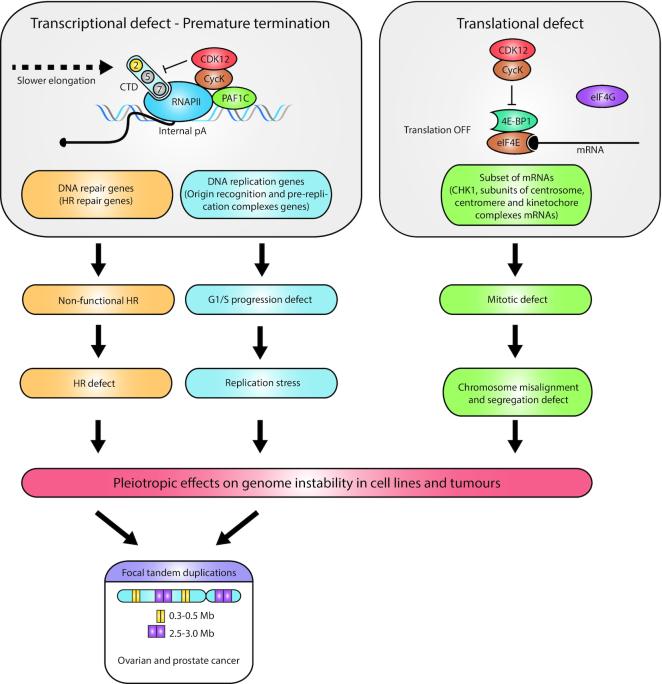
Aberrant CDK12-regulated gene expression has pleiotropic role in onset of genome instability. The schema depicts multiple ways in which non-functional CDK12 causes genome instability. (Left) The premature transcription termination of many HR and DNA replication genes results in an inactive HR DNA repair pathway and G1/S progression defects. The subsequent onset of HR defects and replication stress contributes to genome instability in cells. In ovarian and prostate cancers with inactive *CDK12*, a parallel onset of HR defects and replication stress likely leads to the onset of a unique CDK12-specific genome instability phenotype, focal tandem duplications. (Right) Inactive CDK12 does not allow the release of 4E-BP1 from eIF4E, which leads to suboptimal translation of a subset of mRNAs, mainly CHK1 and several subunits of centrosome, centromere and kinetochore complexes. The subsequent mitotic defect is characterized by chromosome misalignment and segregation defects, which also contribute to cellular genome instability.

The ability of CycK to restore a cell cycle progression deficiency by substituting missing G1 cyclins in *S. cerevisiae* provided the very first hint that a CycK-associated kinase(s) can regulate cell cycle progression (2). In agreement with a role of CycK complexes in the regulation of cell cycle progression were findings that *CycK* is highly expressed in fast-growing stem cells (12) and little in non-proliferative tissues (77). Moreover, THZ531 and ATP analogue 3-MB-PP1 inhibited cellular proliferation in various cell lines and engineered AS CDK12 cells, respectively (38,41). A later study showed the arrest of synchronized cells in the G1 phase upon CycK depletion (78). The proposed mechanism suggested a direct role of the CDK12-dependent phosphorylation of cyclin E1 in the pre-replication complex assembly (independently of CDK12-regulated transcription) (78). Inhibition of the CDK12 in synchronized cells carrying AS CDK12 alleles showed that the kinase activity is required for optimal G1/S progression (39). The inhibition affected the transcription elongation of many origin recognition and pre-replication complexes genes, including *CDC6*,*CDT1*,*TOPBP1* and *MTBP*, resulting in their diminished protein levels, disrupted loading on chromatin, aberrant formation of pre-replication complexes and delay in G1/S progression (39) (Figure 4). These gene groups are regulated by E2F transcription factors (79), but their recruitment to the promoters is not affected by CDK12 inhibition (39). This points to a rate-limiting role of the transcription elongation in the regulation of many DNA replication and repair genes. Importantly, the short CDK12 inhibition also provided strong evidence for the G1/S progression defect being independent of the secondary activation of DNA damage pathways, which occurs well after the cell cycle progression defect (39).

CycK or CDK12 knockdown also leads to an accumulation of cells in G2/M, which was initially interpreted to be a secondary effect of the activation of the DNA damage cell cycle checkpoint (80) after the diminished expression of CDK12-dependent DNA repair genes (5). However, a recent study has shown that CycK depletion also decreases the expression of Aurora B kinase, a key mitotic regulator, and leads to the onset of Aurora B-dependent mitotic catastrophe and G2/M arrest (81). As the translation of many other core mitotic regulatory proteins is critically dependent on CDK12, it can be concluded that aberrant catalytic activity of CDK12 results in defects in multiple steps of mitosis and a severe mitotic defect (74) (Figure 4).

In summary, recent studies provided strong support for several fundamental roles of CycK/CDK12 in the regulation of the cell cycle and cellular proliferation.

## THE REGULATION OF GENOME STABILITY BY CDK12 AND FOCAL TANDEM DUPLICATIONS

As CDK12 directs several cellular processes whose disruption triggers DNA damage, it likely plays a pleiotropic and cellular context-dependent role in the regulation of genome stability (Figure 4). CDK12 regulates the transcription of long DNA repair genes (such as *BRCA1*,*BRCA2*,*ATR*,*ATM*,*Fanconi anaemia*), predominantly those involved in the HR repair pathway (5,10,14,38,39,46,47,82). Thus, alterations in CDK12 generate a non-functional HR pathway, endogenous DNA damage, genome instability and sensitivity to DNA damage agents (5,10,82,83). The depletion or inhibition of CDK12 also results in defects in DNA replication and G1/S progression (39,78), likely leading to replication stress that is another source of genome instability (84,85). Furthermore, disruption of CDK12-dependent translation network of components and regulators of spindle assembly checkpoint (74) also contributes to CDK12-dependent genome instability (86,87).

Analyses of ovarian and prostate tumours with biallelic inactivation of *CDK12* revealed a unique genome instability phenotype characterized by copy-number gains (47,88–92). The focal tandem duplications have a bimodal distribution of ∼0.3–0.5- and ∼2.5–3.0-Mb-long duplicated segments throughout the genome, especially in gene-rich regions (88) (Figure 4). Importantly, the focal tandem duplications are distinct from duplications in *BRCA1*-deficient, *cyclin E1*-amplified and other HR/DNA repair-deficient tumours (47,88,89,91,93). The size of the duplicated segments corresponds to the length of replication domains, which is consistent with their origin in defective DNA re-replication in the S phase (47,88,94). In contrast to the depletion or inhibition of CDK12 in various cancer cell lines, the decreased expression of BRCA1/2 and some other HR factors was not found in ovarian and prostate *CDK12*-inactivated tumour samples (10,47,88,89). Consistently, the tumour gene expression profiles were distinct from HR-deficient tumours (47,88). Thus, the molecular mechanism of the genesis of focal tandem duplications cannot be solely caused by the aberrant expression of CDK12-dependent HR/DNA repair genes. There are numerous possible scenarios. Given that the specific inhibition of CDK12 kinase activity in the cancer cell line leads to the aberrant expression of HR and also many origin recognition and pre-replication complexes genes (39), it is possible that the onset of replication stress and deficient HR-mediated fork restart could lead to their genesis (39,95) (Figure 4). At the same time, a compensatory mechanism for the expression of CDK12-dependent HR genes (particularly *BRCA1/2*) in the tumour background must exist, and might even be essential for tumour onset and/or survival. Notably, *CDK12* mutant prostate cancer tumours exhibit synthetic dependence on recurrent gains in several genes involved in the regulation of the cell cycle and DNA replication such as *MCM7*,*CCND1* or *RAD9A* (88).

In summary, CDK12 inactivation in cell lines leads to the aberrant expression of many genes crucial in various pathways and processes essential for the maintenance of genome stability. However, *CDK12*-inactivated ovarian and prostate tumours present a unique genome instability phenotype, pointing to a unique deregulation of genome stability (likely by aberrant DNA replication-associated HR-dependent repair) leading to tumorigenesis. Interestingly, very recent analyses have revealed the presence of focal tandem duplications in many other cancers with a low incidence (<2%) of *CDK12* inactivation (96), suggesting their occurrence in addition to ovarian and prostate cancers.

## *CDK12* ABERRATIONS IN TUMOURS

Genomic alterations in *CDK12* were documented in ∼30 tumour types with an incidence of up to 15% of sequenced cases (97) with molecular consequences best studied in ovarian, breast and prostate cancers. *CDK12* aberrations in tumours include mutations, deletions, amplifications, rearrangements and overexpression. The aberrations emerge as biomarkers for patient stratification and have the potential to guide therapeutic interventions. CDK12 is both a tumour suppressor and oncogene, and the functional outcomes of *CDK12* aberrations are case and context dependent (46,97,98).

### CDK12 as a tumour suppressor

The tumour-suppressive role of CDK12 is linked to its ability to maintain genome stability via regulating the transcription of DNA repair genes (5,10,14,38,39,46,47,82,99). The roles of CDK12 in the optimal transcription and translation of DNA replication genes (39,78) and mitotic regulators (74), respectively, significantly contribute to the tumour-suppressive function of CDK12. The inactivation of *CDK12* (mutations and deletions in the kinase domain) results in the loss of catalytic activity and tumour-suppressive function of the kinase. The mutations of *CDK12* in high-grade serous ovarian cancer (HGSOC) are mostly homozygous, indicating that they are driver mutations of a tumour suppressor (100).

### CDK12 as an oncogene

Evidence is emerging that CDK12 participates in multiple oncogenic pathways and signalling. In line with the concept of transcriptional addiction (101), CDK12 as a transcriptional regulator of DNA damage and replication genes can improve the fitness of cancer cells, as they are often substantially dependent on the DNA repair system (102). Moreover, the overexpression of c-MYC, a central oncogene driving many tumours (103,104) and super-enhancer-associated transcription factor genes, including *RUNX1* and *MYB* (38), depends on CDK12. *CDK12* is co-located close to the tyrosine kinase receptor *HER2* (also *ERBB2* or *EGFR2*) at locus Ch17q12 (105) and is often co-amplified with this oncogene in *HER2*-positive (amplified) breast cancer (105,106). The resulting increased expression of CDK12 mRNA and protein accompanied by increased phosphorylation of CDK12 was suggested to drive the oncogenic activities of CDK12 in this type of breast cancer (106–109). Indeed, a recent study showed a CDK12-dependent transcriptional upregulation of IRS1 and WNT ligands leading to the activation of oncogenic ERBB–PI3K–AKT and WNT signalling pathways in *HER2*-positive breast cancer (110). It is worth noting that *HER2*-positive breast cancers are mutually exclusive with breast tumours carrying mutations in *BRCA1*, which is consistent with the incompatibility of overexpressed CDK12 during the genesis of HR-defective tumours (106). Another oncogenic property of overexpressed CDK12 leads to the downregulation of the DNAJB6-L protein (via ALE splicing of its mRNA), which promotes the cell migration and invasiveness of *HER2*-positive breast cancer cells (46). The CDK12-specific focal tandem duplications with a high number of duplications and fusions can lead to the differential expression of oncogenic drivers such as *CCND1* or AR (androgen receptor) or c-MYC enhancers as a secondary genetic event of CDK12 inactivation (88,89,111).

## THERAPEUTIC POTENTIAL OF CDK12

The potential of CDK12 as a major therapeutic anti-cancer target is gradually being discovered. Increasing knowledge of *CDK12* aberrations in tumours, the roles of CDK12 in various cellular processes and the recent availability of CDK12 inhibitors have contributed to this exciting development. Numerous studies have started to reveal the cellular and genetic background that determines sensitivity to CDK12 inhibition (Table 1). This includes defects in the HR pathway, MYC overexpression, *HER2* amplification and the expression of EWS/FLI fusion protein. Moreover, functional studies suggest that CDK12-mediated cell cycle and metabolic vulnerabilities and the CDK12-induced neoantigens load might be promising candidates/biomarkers for targeted CDK12-specific cancer therapy (Table 1).

**Table 1.** Overview of therapeutic sensitivity of tumours/tissues with indicated phenotype and *CDK12* status

Tissue/tumour	*CDK12* status	Related phenotype	Drug sensitivity	References
Breast (TNBC)	WT	HR-proficient	CDK12 inhibitors (dinaciclib, SR-4835) + PARP inhibitors (olaparib) or DNA-damaging drugs (platinum-based, doxorubicin, irinotecan)	(42,116)
Ovarian (HGSOC) and breast	Inactivating mutations; shRNA/siRNA depletion of WT	HR deficiency	PARP inhibitors, DNA-damaging drugs	(10,82,83)
Breast (*HER2*-positive)	Genomic disruption (out-of-frame rearrangements due to breakpoint of *HER2* amplicon)	HR deficiency	PARP inhibitors	(108,109)
Metastatic osteosarcoma	WT	HR-proficient	CDK12 inhibitors (dinaciclib, THZ531)	(117)
Foreskin fibroblasts	siRNA depletion of WT	c-MYC overexpression		(103)
Neuroblastoma and ovarian	WT	*N-MYC*/*c-MYC* amplification	CDK inhibitors (roscovitine, CR8, THZ1)	(104,124)
Breast (trastuzumab- resistant/sensitive HER2-positive)	Amplified	*HER2* amplification	CDK12 inhibitor (dinaciclib, THZ531)	(110)
Hepatocellular carcinoma (sorafenib-treated)	WT	EGFR/HER3 and PI3K/AKT activation	CDK12 inhibitor (THZ531)	(126)
Ewing sarcoma	WT	EWS/FLI expression	CDK12 inhibitor (THZ531) + PARP inhibitors (olaparib)	(128)
Cancer cell lines (colon, ovarian)	siRNA depletion of WT	Replication stress	CHK1 inhibitors	(129)
Prostate (mCRPC)	Biallelic loss-of-function mutations	Focal tandem duplications, high neoantigen load	Immune checkpoint (PD-1) inhibitors	(88,94,132)
Ovarian (HGSOC)	siRNA depletion of WT	*BRCA1* deficiency (HR deficiency-independent metabolic reprogramming)	Metabolic inhibitors	(135)

WT = wild-type *CDK12*.

### Tumours with defects in the HR pathway: PARP inhibitors and platinum-based chemotherapies

Tumours sensitive to PARP inhibitors (PARPi) have a non-functional HR pathway due to aberrations in either *BRCA1/2* or other components of the HR pathway (112–114). CDK12 was found to be one of the determinants of PARPi sensitivity in a genome-wide screen (82), consistent with its crucial role in the transcription of many HR genes. About 50% of HGSOC and triple-negative breast cancer (TNBC) are defective in the HR pathway, with *CDK12* being among several recurrently mutated DNA damage response genes (4,115). *CDK12* mutations tend to be mutually exclusive with mutations in other HR genes (82), indicating they are a primary cause of the HR-deficient phenotype. Indeed, *CDK12*-inactivating aberrations found in HGSOC failed to support HR repair (10) and sensitized the cells to PARPi and platinum-based drugs (82,83). In a subset (∼14%) of *HER2*-positive breast cancer, the *HER2* amplicon breakpoint converges on *CDK12*, disrupting its expression and leading to sensitivity to PARPi (108,109). Dinaciclib, a potent CDK12 inhibitor, reversed PARPi resistance in models of TNBC with mutated *BRCA1/2*, pointing to a possibility of the combinatorial use of CDK12 and PARP inhibitors (116). Likewise, treatment with SR-4835 in HR-competent TNBC led to the common suppression of DNA repair genes and synergistic promotion of sensitivity to PARPi and to various DNA-damaging agents, including cisplatinum, doxorubicin and the topoisomerase inhibitor irinotecan (42). In metastatic osteosarcoma (OS), a cancer with a high degree of genome instability, treatment with several CDK12 inhibitors led to the sensitivity of OS cell lines and a decrease in metastatic cell outgrowth in the lungs, perhaps partly due to the defective expression of DNA damage response genes (117).

Overall, since multiple PARPi have been approved for clinical use in breast and ovarian cancers with *BRCA1/2* mutations (118), *CDK12* mutations could be used as other biomarkers for their application. Moreover, CDK12 inhibitors have the potential to be used to reverse resistance to PARPi in tumours with residual HR activity and to enhance the efficiency of existing DNA-damaging drugs, including widely used platinum-based chemotherapies.

About 7% of metastatic castration-resistant prostate cancers (mCRPCs) carry aberrations in *CDK12* and ∼24% have a non-functional HR pathway (88,119,120). This suggests sensitivity to PARPi in a considerable proportion of mCRPCs, including those with *CDK12* aberrations. However, when compared to other HR-deficient mCRPCs, the *CDK12*-abberant tumours were found to be transcriptionally, genetically and phenotypically different (88) and displayed more aggressive clinical behaviour (including higher Gleason scores at presentation, a shorter time to metastasis and CRPC) (121). This indicates that they represent a molecularly distinct subtype of mCRPC with potential for a different and/or more intensive therapeutic approach (88,121). Indeed, preliminary results of a clinical trial with PARPi rucaparib in mCRPC patients with HR gene mutations revealed that none of the patients with *CDK12* alterations exhibited a response to the treatment, in contrast to patients with *BRCA1/2* mutations (122). This suggests that the response of cancers with *CDK12* aberrations to PARPi may be tumour type and context specific. It also points to the need for a more elaborate stratification of patients with HR-deficient tumours and the application of alternative treatments, such as immunotherapy as discussed later, for certain groups of patients with *CDK12* aberrations.

### Tumours with MYC overexpression

The MYC family of transcription factors is deregulated in >50% of human cancers. In normal cells, their expression is tightly regulated; in tumours, they are often amplified and overexpressed, and MYC-driven transcription programmes are central drivers of the disease. MYC proteins are considered directly ‘undruggable’ (123), but their overexpression is dependent on other regulators, including various transcription elongation factors that are druggable and provide a therapeutic opportunity (97,101,103). Indeed, c-MYC overexpression in fibroblasts is synthetically lethal with CDK12 depletion (103). The application of inhibitors that target multiple CDKs, including CDK12, led to the downregulation of N-MYC and c-MYC and their transcription programmes in MYC-driven neuroblastoma and ovarian cell lines, respectively (104,124). Additionally, CDK12 cooperates with c-MYC to promote the mTORC1-dependent translation of mRNAs of several oncogenic factors (74). In summary, the inhibition of CDK12 seems to be a promising tool for the treatment of various MYC-dependent cancers.

### Tumours with *HER2* amplification

*HER2*-positive breast cancers are treated with anti-HER2 monoclonal antibodies such as trastuzumab (Herceptin), but >50% of patients develop resistance (125). A recent study has suggested that the resistance is induced via the CDK12-mediated overexpression of several WNT ligands and components of the ERBB–PI3K–AKT pathway including IRS1, which leads to the activation of the pro-growth signalling cascades (110). Consistently, the pharmacological inhibition of CDK12 exhibited anti-proliferative effects in trastuzumab-resistant but also trastuzumab-sensitive cells, suggesting that CDK12 inhibition might serve as a replacement therapy for trastuzumab in breast cancers with amplified *HER2* and *CDK12* (110). Interestingly, adaptive response to sorafenib, an anti-hepatocellular carcinoma drug, is caused by the aberrant activation of EGFR/HER3 receptors and the PI3K/AKT pathway, and was reversed by THZ531 treatment (126). This suggests an exciting possibility of targeting these oncogenic pathways by CDK12 inhibition in various types of tumours.

### Tumours expressing EWS/FLI fusion protein

EWS/FLI fusion protein is a transforming transcriptional activator in Ewing sarcoma and currently difficult to target (127). CDK12 inhibition in Ewing sarcoma is synthetically lethal with EWS/FLI expression and leads to the downregulation of DNA repair genes (128). In this genetic and cellular context, THZ531 also exhibited a synergistic effect with PARPi and various DNA-damaging drugs (128). Thus, the interference with CDK12′s roles as transcriptional co-activator and master regulator of DNA damage response genes suggests a promising translational potential for the treatment of this disease.

### CHK1 and inhibitors of cell cycle checkpoints

Recent findings that CDK12 (directly or indirectly) regulates various steps of cell cycle progression (39,78,81) provide fresh avenues to identify and exploit other synthetically lethal interactions of CDK12 in various genetic and cellular contexts. Notably, cells with depleted CDK12 were shown to be more reliant on the kinase activity of CHK1 (129). Further research in this field is warranted and will likely provide more translation opportunities for the treatment of various malignancies.

### Immunotherapy-immune checkpoint inhibitors

Immune checkpoint blockage with PD-1 inhibitors is an emerging strategy for the treatment of cancer. A subgroup of mismatch repair (MMR)-deficient tumours with high neoantigen load and T-cell infiltration is highly sensitive to the therapy (130,131). Remarkably, 7% of mCRPC carrying a biallelic inactivation of *CDK12* and focal tandem duplications have increased gene fusion, fusion-induced neoantigen load and T-cell infiltration, suggesting that this new subgroup of mCRPC could also benefit from the treatment (88). This was also indicated by a small pilot clinical study (88) and recently confirmed by a much larger clinical study (132). As the biallelic loss of *CDK12* is common in other types of tumours (133,134) and in some of them is associated with focal tandem duplications (96), the *CDK12* aberrations have a potential to be used, next to MMR deficiency, as another biomarker of response to the therapy (94).

### Inhibition of energy metabolism

A recent study has shown that reducing BRCA1 expression, either directly or via CDK12 depletion, led to HR deficiency-independent metabolic reprogramming and an increased sensitivity of *BRCA1*-deficient HGSOC cells to metabolic inhibitors (135). This finding raises the question of whether tumours with *CDK12* aberrations have metabolic vulnerabilities that could be targeted by metabolism-modulating drugs (136).

## CLINICAL TRIALS

There are ongoing clinical trials on patients with *CDK12*-aberrant tumours. Selected examples are shown in Table 2.

**Table 2.** List of sample clinical trials on patients with *CDK12*-aberrant tumours

Tumour type	Condition	Intervention/treatment	Identifier/study abbreviation
mCRPC	Loss of *CDK12* function	Checkpoint inhibitor immunotherapy (nivolumab: anti-PD-1; ipilimumab: anti-CTLA-4)	NCT03570619, IMPACT
mCRPC	MMR deficiency or biallelic inactivation of *CDK12*	Checkpoint inhibitor immunotherapy (pembrolizumab: anti-PD-1)	NCT04104893, CHOMP
mCRPC	HR deficiency	PARPi (rucaparib)	NCT02952534, TRITON2
Renal cell carcinoma	Inactivating mutations in *CDK12* (or other DNA repair genes)	PARPi (olaparib)	NCT03786796, ORCHID
mCRPC	Mutations in non-canonical DNA repair genes including *CDK12*	PARPi (olaparib)	NCT03012321
mCRPC	Positive for DNA repair gene defects or *CDK12* biallelic inactivation	PARPi (niraparib) combined with checkpoint inhibitor immunotherapy (cetrelimab: anti-PD-1)	NCT03431350, QUEST

Source: ClinicalTrials.gov (10 February 2020).

## SUMMARY AND FUTURE DIRECTIONS

Over the last few years, our knowledge of CDK12 has expanded dramatically and its medical potential is being realized. Nevertheless, many questions about its roles and functions in the cell remain open.

Mechanistically, it will be important to determine which other proteins mediate CDK12 recruitment to genes, and which residues and repeats in the CTD of RNAPII and other cellular substrates are phosphorylated by CDK12. This knowledge will be critical to deciphering the precise mechanism of CDK12-dependent transcription elongation and for elucidating why some genes are more dependent on CDK12 than others (not all long, polyadenylation-site-rich genes are dependent on CDK12 and vice versa). Another important question is whether the inhibition of premature termination by CDK12 is a regulated or merely a passive process. Given the occupancy of CDK12 on gene promoters, its potential role in promoter-proximal pause release and re-initiation remains to be determined. Broader questions of high interest include determining how exactly CDK12-directed gene expression is integrated into the regulation of DNA replication and cell cycle progression as well as determining the mechanism of genesis of focal tandem duplications.

Answers to these questions will move us forward in understanding the cellular functions of CDK12. They will likely create fresh avenues towards clinical applications, such as finding new synthetic lethal interactions and its use as a biomarker for treatments of various malignancies.
